# A FRAMEWORK FOR UNDERSTANDING PALLIATIVE CARE PREFERENCES AMONG AFRICAN AMERICAN PATIENTS WITH CANCER: THE 6S MODEL

**DOI:** 10.1093/geroni/igae098.3775

**Published:** 2024-12-31

**Authors:** Elisha Oduro, Joan Carpenter

**Affiliations:** University of Maryland, Baltimore, Baltimore city, Maryland, United States; University of Maryland, Baltimore, Baltimore, Maryland, United States

## Abstract

African American (AA) patients with cancer have the highest mortality rates and poorest outcomes among all racial groups in the US, yet they are almost one-third less likely to receive palliative care (PC) than their White counterparts. AAs are less likely to receive PC due to systemic racism, discrimination, provider bias, and culturally insensitive healthcare services. Additionally, research has primarily focused on highlighting the disparities in PC access among AA patients with cancer rather than understanding the experiences and preferences of those who do receive PC services. Grounded in 6S-Model for Person-Centered Palliative Care, this presentation aims to illustrate a theoretically derived approach for clinicians and researchers to develop culturally sensitive PC for AA patients with cancer. The model comprises six concepts elucidating different aspects of patient care to ensure sensitive care and overcome disparities: self-image, symptom relief, social relations, self-determination, synthesis, and strategies. Self-image is the model’s central concept, reflecting the patient’s personal experiences and identity. To preserve self-image, it is crucial to alleviate the patient’s symptoms and physical suffering while maintaining their social connections. Self-determination ensures patient involvement in their care, and synthesis and strategies address their care preferences, spiritual and existential needs. Utilizing the 6S Model for Person-Centered Palliative Care in studies and clinical care, will allow AA patients with cancer to share their PC experiences and collaborate in creating culturally sensitive PC, thereby integrating AA patients with cancer preferences and needs into PC, improving quality of life, care outcomes, and promoting equity in PC services.

